# Stomach-Worm (*S. Contortus*, Rud.) in Lambs

**Published:** 1900-05

**Authors:** A. G. Hopkins

**Affiliations:** Assistant in Animal Husbandry, Agricultural Experiment Station, University of Wisconsin


### STOMACH-WORM (*S. CONTORTUS*, RUD.) IN LAMBS.

By A. G. Hopkins, D.V.M., B. Agr.,
ASSISTANT IN ANIMAL HUSBANDRY, AGRICULTURAL EXPERIMENT STATION, UNIVERSITY OF WISCONSIN.

In sheep-raising districts the stomach-worm (*S. contortus*, Rud.) has proved a serious hinderance to successful sheep husbandry. Numerous breeders have had losses of lambs during the past few summers with mortality rates in their flocks of from 15 to 60 per cent. Such being the case, an experiment was conducted at this station to determine the effectiveness of one or two remedies. Benzine had been highly recommended, so this medicament was tried as was creolin (Merck-Pearson). An experiment was under way at the station to determine the relative merits of various grains for feeding, so that the frequent weighings at regular intervals assisted in the diagnosis. Post-mortems were also held, and the stomach-worm found in the abomasum. In one case a tapeworm (*T. expansa*, Rud.) sixteen feet in length was found in the intestines; in one other evidences of nodular disease (*Esophagostoma columbiana*, Curtice) were quite plain. Outside of these two cases the *S. contortus* seemed to be the causative factor of the rapid emaciation and loss of weight, dulness, hydræmia, anorexia, pyrexia, pica, coughing, and frequent micturition, during which large quantities of colorless urine were passed. Intense thirst was shown, lambs affected rushing to the water-trough immediately on being brought in from the pasture. Diarrhœa was not a symptom in the station flock, but has been reported in other flocks quite often.

A tonic powder of ferrisulph., nut areca, nux vomica, and red gentian was administered with practically no results. The lambs were then divided into two lots (I. and II.). Lot I. was given benzine, ʒij, in new milk, ℥ij, on three successive mornings. Lot II. was given creolin, ʒj, in new milk, ℥ij, also on three successive mornings, the medicine being administered fasting. On the fourth morning ol. lini (raw), ℥iij, was administered to each lamb. The next weighing (some ten days later, taking place every two weeks) showed gains of from two to five pounds, which gains were continued, judged by subsequent weighings. Previous to the treatment the lambs were losing weight or else standing still. The medicine was administered as a drench by the station shepherd. Great care is necessary in drenching lambs, and unless in experienced hands fatalities often occur. To the novice a safe method is recommended—namely, a piece of rubber tubing and funnel. The tubing is slowly introduced into the œsophagus and the medicine put into the tubing *via* the funnel. The treatment was given early in September, which is quite late in the season. Prophylaxis, by administering the aforesaid remedy to all lambs in the flock in July or August, will afford the most satisfactory method of controlling this very serious scourge. Benzine (gasoline is said to be equally effective) is probably the better remedy, owing to its cheapness and its palatability as compared with creolin. Worm powders are comparatively worthless after a certain stage. One farmer reports a loss of one hundred and eighty lambs out of a flock of four hundred, in spite of the use of one hundred pounds of some patent worm powder.

